# A correlational study of Weifuchun and its clinical effect on intestinal flora in precancerous lesions of gastric cancer

**DOI:** 10.1186/s13020-021-00529-9

**Published:** 2021-11-20

**Authors:** Yanqin Bian, Xi Chen, Hongyan Cao, Dong Xie, Meiping Zhu, Nong Yuan, Lu Lu, Bingjie Lu, Chao Wu, Nisma Lena Bahaji Azami, Zheng Wang, Huijun Wang, Yeqing Zhang, Kun Li, Guan Ye, Mingyu Sun

**Affiliations:** 1 Key Laboratory of Liver and Kidney Diseases (Ministry of Education), Institute of Liver Diseases, Shuguang Hospital, Shanghai University of Traditional Chinese Medicine, 528 Zhangheng Road, Pudong New District, Shanghai, 201203 China; 2 Arthritis Institute of Integrated Traditional Chinese and Western Medicine, Shanghai Academy of Traditional Chinese Medicine, Guanghua Hospital of Integrated Traditional Chinese and Western Medicine, Shanghai University of Traditional Chinese Medicine, Shanghai, 200052 China; 3 Department of Infectious Disease and Gastroenterology, Shanghai TCM-Integrated Hospital, Shanghai University of Traditional Chinese Medicine, Shanghai, 200080 China; 4 Department of Gastroenterology, Shuguang Hospital, Shanghai University of Traditional Chinese Medicine, Shanghai, 201203 China; 5 Central Research Institute, Shanghai Pharmaceuticals Holding Co., Ltd., Building 4, No. 898, Halei Road, Pudong New Area, Shanghai, 201203 China

**Keywords:** Precancerous lesions of gastric cancer, Intestinal microbiota, Weifuchun, Randomized controlled clinical trial

## Abstract

**Background:**

Weifuchun (WFC), a Chinese herbal prescription consisting of Red Ginseng, Isodon amethystoides and Fructus Aurantii, is commonly used in China to treat a variety of chronic stomach disorders. The aim of the paper was to determine the effect of WFC on intestinal microbiota changes in precancerous lesions of gastric cancer (PLGC) patients.

**Methods:**

PLGC patients of *H. pylori* negative were randomly divided into two groups and received either WFC tablets for a dose of 1.44 g three times a day or vitacoenzyme (Vit) tablets for a dose of 0.8 g three times a day. All patients were treated for 6 months consecutively. Gastroscopy and histopathology were used to assess the histopathological changes in gastric tissues before and after treatment. 16S rRNA gene sequencing was carried out to assess the effects WFC on intestinal microbiota changes in PLGC patients. Receiver Operating Characteristics (ROC) analysis was used to assess the sensitivity and specificity of different intestinal microbiota in distinguishing between PLGC patients and healthy control group.

**Results:**

Gastroscopy and histopathological results indicated that WFC could improve the pathological condition of PLGC patients, especially in the case of atrophy or intestinal metaplasia. The results of 16S rRNA gene sequencing indicated that WFC could regulate microbial diversity, microbial composition, and abundance of the intestinal microbiota of PLGC patients. Following WFC treatment, the relative abundance of *Parabacteroides* decreased in WFC group when compared with the Vit group. ROC analysis found that the *Parabacteroides* could effectively distinguish PLGC patients from healthy individuals with sensitivity of 0.79 and specificity of 0.8.

**Conclusions:**

WFC could slow down the progression of PLGC by regulating intestinal microbiota abundance.

*Trial registration* NCT03814629. Name of registry: Randomized Clinical Trial: Weifuchun Treatment on Precancerous Lesions of Gastric Cancer. Registered 3 August 2018-Retrospectively registered, https://register.clinicaltrials.gov/ NCT03814629.

## Background

Among all cancers, gastric cancer ranks fifth in terms of incidence and third in terms of mortality worldwide [1]. More than 70% of new gastric cancer cases are found in developing countries. In addition, 42.6% of the global incidence and 45% of all gastric cancer-related deaths occur in China [2], which is attributed to the low screening and diagnosis rates of early gastric cancer. Early detection of precancerous lesions of gastric cancer (PLGC) to halt their further development can effectively reduce the incidence of gastric cancer.

Recent research has established that gastric cancer is associated with bacterial dysbiosis within the stomach, especially *Helicobacter pylori * (*H. pylori*) in the stomach*.* There is increasingly compelling evidence that the microbiome can affect gastrointestinal carcinogenesis [3], especially gut microbiota, which plays a role in gastric cancer formation, development and response to treatment [4]. Recent advances in metagenomics and bioinformatics have provided new insights on the microbial ecology in gastric tumor. By using advanced sequencing technology, more intestinal flora involved in gastric cancer occurrence and cancer treatment can be found.

So far, no one drug has been shown to be effective in treating PLGC with the exception of anti-*H. pylori* therapy. In China, vitacoenzyme tablets were approved for the treatment atrophic gastritis and esophageal epithelial hyperplasia. The tablets are a compound preparation from plant Soybean, and their main components are riboflavin and riboflavin derivatives. In fact, vitacoenzyme was included in several studies involving chronic atrophic gastritis (CAG) [5] and gastric precancerous lesions(GPL) [6, 7]. These studies found that vitacoenzyme had limited effect in protecting gastric mucosa against CAG.

Weifuchun (WFC) tablet is a well-known Chinese herbal drug, which was approved by China food and drug administration (CFDA) in 1982. WFC became a patent drug in 1995. It is composed of three herbs, Renshen (Red Ginseng), Xiangchacai (Isodon amethystoides) and Zhike (Fructus Aurantii), and has the effects of strengthening the spleen and replenishing qi, promoting blood circulation and detoxification, eliminating gas and phlegm [8]. Currently, WFC has been widely used in the treatment of a variety of chronic stomach disorders including CAG and GPL [9, 10]. Annual sales of WFC are 260 million US dollars in 2018. Recent studies showed that WFC could inhibit inflammation of *H. pylori* infected gastric epithelial cells by blocking NF-kappaB pathways [11]. In our previous research, we found that WFC could inhibit inflammation and increase pepsin secretion by inhibiting MAPK signaling pathway [12]. Studies also demonstrated that WFC was antispasmodic and analgesic, and its functions included regulating gastrointestinal motility [13], inhibiting gastric acid secretion, protecting the gastric mucosa [13, 14], and improving histological endoscopic findings and symptoms [10] of PLGC.

However, there is not enough evidence in clinical trials regarding WFC ability to relieve PLGC and its mechanism is still unknown. More large scale randomized and control trials are needed to investigate WFC’s effectiveness on PLGC. In this study, we evaluated WFC’s effect on PLGC and assessed histopathological changes using a randomized and controlled trial. The stool samples of patients were collected to analyze the intestinal microbial abundance by high-throughput sequencing 16SrRNA. The results elucidate the probable mechanism of action of WFC in regulating intestinal microbial balance and treating atrophy and intestinal metaplasia(IM) to alleviate PLGC.

## Methods

### Quality and quantity analyses of Weifuchun

WFC tablet was kindly provided by Huqingyu-tang Pharmaceutical Co., Ltd. (Hangzhou, China). Quality and quantity analyses of the aqueous extract were performed with UPLC TOF-MS. HPLC-grade acetonitrile, methanol, and formic acid were purchased from Fisher Scientific (Santa Clara, USA). Naringin, ginsenoside Rb1, and oridonin were identified in WFC by UPLC TOF-MS. The following conditions were used to analyze naringin, ordionin, and ginsenoside Rb1: system, Acquity UPLC system (Waters, USA), which consists of a solvent degasser, a binary pump, an auto-sampler and a column oven; column, Acquity UPLC BEH C18 RP column (1.7 μm, 100 mm × 2.1 mm i.d.; Waters, USA); mobile phase A, 0.1% formic acid in water; mobile phase B, 100% acetonitrile; flow rate, 0.3 mL/min; wavelengths, 210 nm for ginsenoside Rb1, 254 nm for ordionin and 280 nm for naringin; injection volume, 10 μL; MS/MS detector, Acquity Synapt G2 Q-TOF tandem mass spectrometer connected to the UPLC system by an ESI interface and controlled by MassLynx version 4.1 (Waters, UK). Samples were analyzed in the positive model. Data were collected and analyzed by Waters MassLynx version 4.1.

### Trial oversight

This randomized and controlled trial was conducted in the outpatient clinics of Shuguang Hospital and Shanghai TCM-Integrated Hospital affiliated to Shanghai University of Traditional Chinese Medicine. All subjects (patients and health volunteers) provided written informed consent before enrollment. The trial was approved by the institutional review board at Shuguang Hospital and was conducted in accordance with the provisions of the Declaration of Helsinki and the CONSORT guidelines. An independent data and safety monitoring board reviewed the progress of the trial.

The study protocol, which describes the study in more detail, can be found in the clinical trial registry (https://register.clinicaltrials.gov) with the identifier NCT03814629. The study was approved by the Ethics Committees of Shuguang Hospital affiliated to Shanghai University of Traditional Chinese Medicine (No. 2016-478-29-01). Recruitment and data collection occurred between October 2015 and September 2017. Patients with a previous histological diagnosis of CAG with or without IM/dysplasia were selected as potential subjects. Health volunteers were those never had stomach trouble and other serious diseases. The trial was not registered until all the patients had been enrolled because registration was not mandated after the trial had started.

### Participants and eligibility criteria

Only those who fulfilled the diagnosis of both CAG and IM or dysplasia according to diagnosis criteria [15] and *H. pylori*(-) were considered eligible subjects, male or female, between 18 and 70 years. Participants with *H. pylori* positive infection without radical treatment, peptic ulcer or severe dysplasia (suspected malignant transformation), severe systemic diseases such as cardiovascular and cerebrovascular disease, hepatic diseases, kidney or lung disease, or with other tumors, were excluded. Participants were excluded if they had an allergic constitution or allergies to any known ingredients in WFC. Finally, patients with other diseases interfering with the study or patients unwilling to undergo repeated endoscopy after treatment were also not included.

The TCM standard for diagnosing syndromes was worked out with reference to the standard for diagnosing the type of spleen and stomach deficiency in the guidelines of diagnosing and treating CAG. Major symptoms were stomach pain or discomfort or stomach symptoms remission after warm or press operation. Minor symptoms included: (1) anorexia; (2) loose stools; (3) physical and mental fatigue; (4) shortness of breath and lazy speech; (5) stomach distention after eating; (6) belching; (7) chest distress; (8) stomach pain and fear of being pressed; and (9) light-colored tongue with small and wiry pulse. Patients with one of the major symptoms and two or more minor symptoms were diagnosed as suffering from the syndrome of spleen and stomach deficiency.

### Trial design and treatment

Before endoscopy, patients were randomly assigned in a 1:1 ratio to receive either WFC therapy or vitacoenzyme. Computer-generated randomization was performed in a blinded manner, with status concealed from all the patients and the primary physician, endoscopist, pathologist, and statistician. After randomization, endoscopy was performed. The patients started the assigned trail medication within 1 week after endoscopy.

Each subject received either WFC tablets (1.44 g) (Hangzhou huqingyutang pharmaceutical co. LTD. Hangzhou, China, lot number 16066129) or vitacoenzyme tablets (0.8 g) (Beihai sunshine pharmaceutical co. LTD, Guangxi, China, lot number 102029), taken orally after meal 3 times a day for 6 months. Before randomization, *H. pylori* status was determined by rapid urease test (RUT) or by pathology. Positive subjects received standard eradication therapy. *H. pylori* status was re-evaluated at the end of the 6th month. If required, the status would be re-evaluated by 13C-urea breath test at 4 weeks after the cessation of therapy.

### Screening measures

The demographics of participants were collected, including age, gender, course of disease, and current and past gastric disease treatment. The histologic diagnosis and grading were made according to the updated Sydney system [16].

### Histological scores

The criteria to evaluate the histopathology were made according to the updated Sydney system [16]. Each gastric tissue was evaluated separately for the following items: chronic inflammation (CI), acute inflammation (AI), atrophy, IM, and dysplasia. Atrophy was defined as loss of glands and graded as absent (0), mild (1), moderate (2), or severe (3). IM was graded as absent (0), mild (1), moderate (2), or severe (3) according to the proportion of the gastric mucosa replaced by the metaplastic tissue. Presence and severity of dysplasia defined by atypical cytological and architectural derangement, subcategory of mild (1), moderate (2), and severe (3) grade adhered to the diagnosis for gastric neoplasia.

### High-throughput sequencing

Total DNA was extracted using the QIAamp DNA Stool Mini Kit. All extractions yielding > 2 ng/μ of total DNA, as indicated by NanoDrop 2000 UV–Vis Spectrophotometer measuring. Each DNA sample was amplified for the V3 region of 16S rRNA gene and libraries were sequenced in a single run of the Illumina MiSeq sequencing platform at NovelBio Biomedical technology Co.,LTD.

### OTU clustering

Bacterial 16S rRNA reads were analyzed using the Quantitative Insights into Microbial Ecology(QIIME) software package [17]. Operational taxonomic units(OUT) were created by single-linkage clustering the reads using Swarm [18] and removing OTUs comprised of only a single or pair of reads. Representative sequences from each OUT were aligned using the PyNAST aligner [19]. All OTUs were tested for correlations between the proportional abundance of OUT and the post-PCR amplicon concentration of a sample according to the methods listed elsewhere [20].

### Statistical analysis

According the clinical report and previous research results [9, 21], we calculated the sample size of 35 patients in each group of the trial population. We allowed for a 15% initial dropout rate, and a further 10% loss to follow-up, resulting in the enrollment of 47 patients in each group. An interim analysis was not planned.

The statistical analyses were performed by using IBM SPSS Statistics 21.0. Data were expressed as mean ± standard or median (range) for continuous variables, and frequencies (percentages) for categorical variables. Student's t-test or Mann–Whitney test or Chi square test was used to compare baselines including demographic data and basic evaluating variables. For comparison of variations from baseline to endpoint, paired t-test was performed on variables with normal distributions, and wilcoxon signed-ranks test on non-normal variables. ANOVA test was used to compare microbial abundances between groups. Also, Chi-square test or Fisher's exact test was used for atrophy and intestinal metaplasia disappearance rate and symptom disappearance rate. All statistical tests were two-sided and assumed to be statistically significant at a level of P < 0.05.

## Results

### Naringin, ginsenoside Rb1, and oridonin contents in the WFC formula

Naringin, ginsenoside Rb1, and oridonin are the major constituents of aqueous extract of *Radix Ginseng Rubra*, *Fructus aurantii*, and *Isodon amethystoides*, respectively. The mass-to charge ratios (m/z) 581.1870, 1109.6107 in positive model for naringin and ginsenoside Rb1, and 363.1826 in negative model for oridonin, were observed in the peaks, confirming that they were protonated forms of naringin, ginsenoside Rb1, and oridonin, respectively.

### Baseline characteristics of participants

**Fig. 1:**
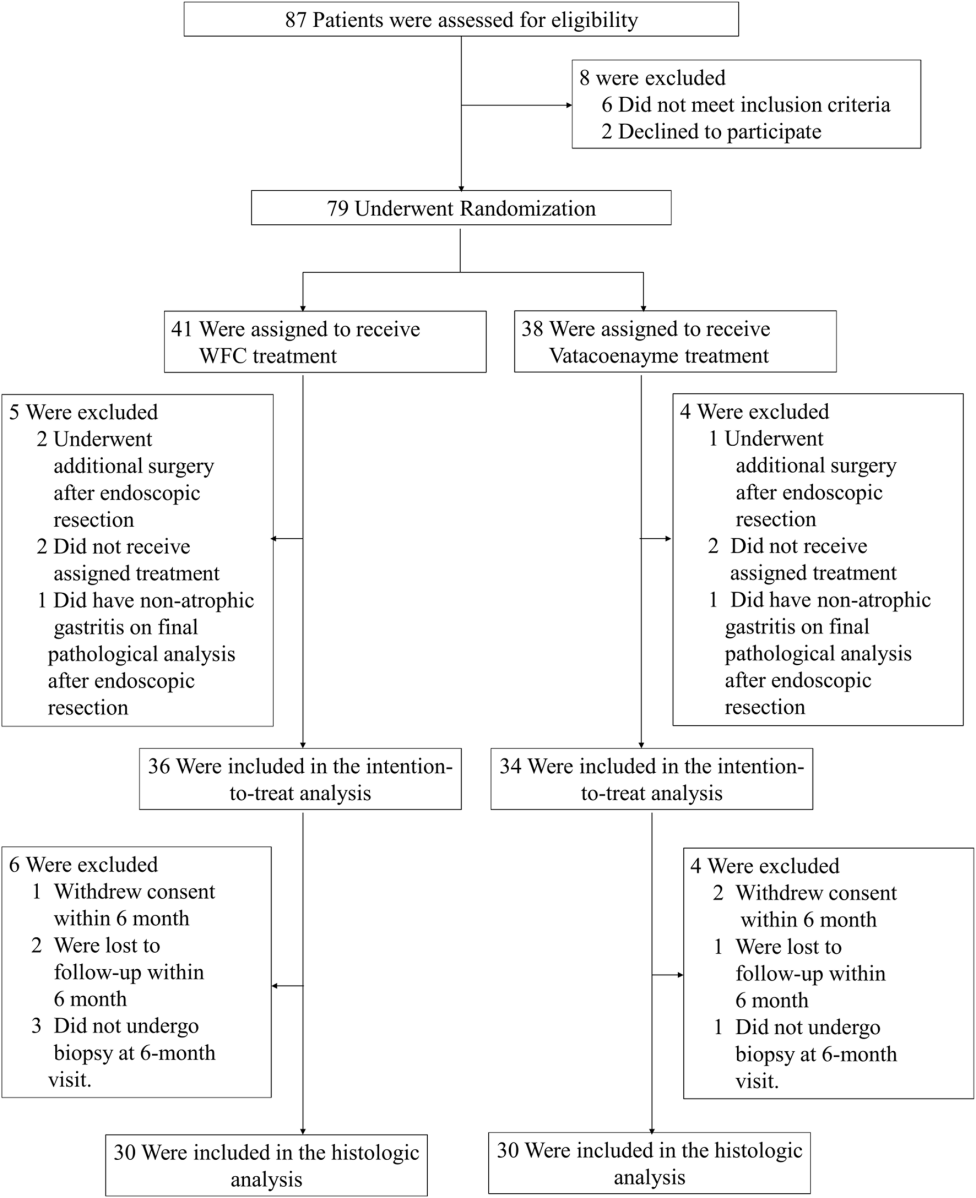
Enrollment, Randomization, and Follow-up. Improvement from baseline in the grade of histopathology detected on gastroscopy at the 6-month follow-up was evaluated in the intention-to treat population. Only patients with gastric tissue specimens obtained at the 6-month follow-up were included in the histologic analysis

Of the 87 patients who were screened, a total of 79 patients underwent randomization (Fig. 1). Of these patients, 70 were included in the intention-to-treat population (36 in the WFC treatment group and 34 in the vitacoenzyme group) after the exclusion of 9 patients, including 3 who underwent additional surgery after endoscopic resection, and 4 who did not receive assigned treatment, and 2 who did not meet other eligibility requirements. Demographics (age, gender and course of disease), histopathology (histological score) and clinical symptom (aggregate score) were similar in the two groups (Table 1). We included 60 patients who had undergone gastroscopy at 6-month follow-up in the histologic analysis.

**Table 1:** Baseline characteristics of participants (*n* = 60)

	WFC (n = 30)	Control (n = 30)	Total (n = 60)	X^2^	t	*P*
Age^a^	50.37 (12.50)	53.90 (10.61)	52.13 (11.53)		1.2	0.243
Sex						
Male	16 (53.33)	15 (50.00)	31 (51.67)	0.268		0.796
Female	14 (46.67)	15 (50.00)	29 (48.33)			
Course of disease^a^	5.57 (4.94)	5.87 (5.10)	5.72 (4.93)		0.2	0.818
Pathology						
Chronic Gastritis^b^						
Mild	7 (23.33)	10 (33.33)	17 (28.33)	1.888		0.596
Moderate	20 (66.67)	19 (63.33)	39 (65.00)			
Severe	2 (6.67)	1 (3.33)	3 (5.00)			
Active Gastritis^b^						
Mild	11(36.67)	7 (23.33)	18 (30.00)	1.765		0.414
Moderate	3 (10.00)	2 (6.67)	5 (8.33)			
Severe	0 (0.00)	0 (0.00)	0 (0.00)			
Atrophy^b^						
Mild	14 (46.67)	19 (63.33)	33 (55)	3.000		0.254
Moderate	12 (40)	10 (33.33)	22 (36.67)			
Severe	4 (13.33)	1 (3.33)	5 (8.33)			
Intestinal metaplasia^b^						
Mild	16 (53.33)	21 (70.00)	37 (61.67)	2.739		0.493
Moderate	10 (33.33)	7 (23.33)	17 (28.33)			
Severe	3 (10.00)	2 (6.67)	5 (8.33)			
Dysplasia^b^						
Mild	6 (20.00)	4 (13.33)	10 (16.67)	2.632		0.235
Moderate	0 (0.00)	0 (0.00)	0 (0.00)			
Severe	0 (0.00)	0 (0.00)	0 (0.00)			
Aggregate score^c^	5.70 (2.00)	4.97 (1.63)			1.6	0.125
Clinical Symptoms						
Aggregate score^c^	15.32 (5.71)		15.92 (5.43)		3.5	0.929

^a^Years, mean(SD)

^b^The number of cases meeting the diagnosis were counted and showed as cases (percent); absent cases were included when P value were calculated through two-sided *X*^2^ test

^c^Aggregate score represented sum of all grade score including absent, mild, moderate and severe; absent, 0; mild, 1; moderate, 2; severe, 3

### Histology and clinical symptom

**Fig. 2:**
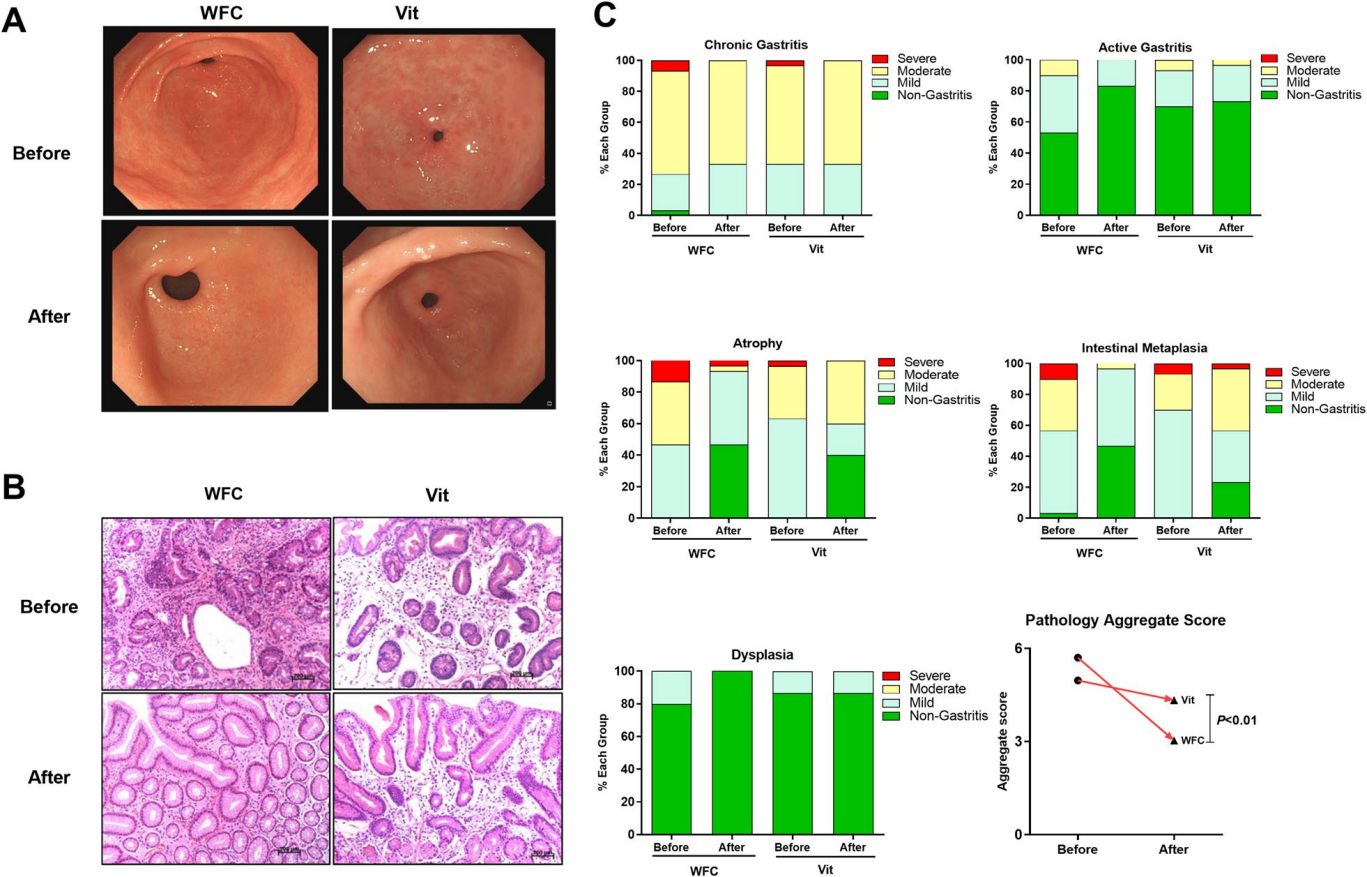
Histopathological Variation. **A** Endoscope variation. **B** Pathological staining in HE. **C** Pathological evaluation. WFC, Weifuchun Group; Vit, vitacoenzyme Group

After treatment, the results of gastroscopy and histopathology improved both in the WFC group and in the vitacoenzyme group (Vit group), especially with regard to atrophy and IM (Fig. 2A–C). A large number of neat glands, less IM, reduced intercellular congestion edema and inflammatory cell infiltration was observed in histopathological findings in WFC group compared with the Vit group (Fig. 2B). The total change value of pathology aggregate score in WFC group remarkably increased compared with the Vit group (Fig. 2C). Patients with gastric mucosal atrophy or with IM in mild grade or moderate grade were the majority before treatment. But after treatment, patients with non-gastritis (normal) grade or mild grade in the WFC group were more than those in the Vit group, suggesting that WFC could improve atrophy and IM. The total effective rate for alleviating atrophy degree was 80% in the WFC group and 23.33% in the Vit group, respectively. And the total effective rate for alleviating IM degree was 73.33% in the WFC group and 26.67% in the Vit group, respectively (total effective rate of alleviating the atrophy and IM degree = the alleviated atrophy and IM degree/ total cases × 100%) (shown in Tables 2 and 3).

**Table 2:** Comparison of gastric mucosal atrophy level

Group	*n*	Atrophy level (*n*)				Change of atrophy level after treatment (*n*)				Effective rate (%)
		Non-Gastritis	Mild	Moderate	Severe	3 Levels	2 Levels	1 Level	0 Level	
WFC										
WFCB	30	0	15	11	4	1	5	18	6	80^**^
WFCA	30	14	14	2	0					
Vit										
WitB	30	0	16	11	3	0	2	5	23	23.33
WitA	30	4	12	12	2					

WFCB, patients before treatment with WFC; WFCA, patients after treatment with WFC; VitB, patients before treatment with vitacoenzyme; VitA, patients after treatment with vitacoenzyme; **, *P* < 0.01, compared with Vit

**Table 3:** Comparison of gastric mucosal intestinal metaplasia level

Group	*n*	Intestinal metaplasia level (*n*)				Change of intestinal metaplasia level after treatment (*n*)				Effective rate (%)
		Non-gastritis	Mild	Moderate	Severe	3 levels	2 levels	1 level	0 level	
WFC										
WFCB	30	1	16	10	3	2	2	18	8	73.33^**^
WFCA	30	14	15	1	0					
Vit										
VitB	30	1	17	10	2	0	1	7	22	26.67
VitA	30	5	10	14	1					

WFCB, patients before treatment with WFC; WFCA, patients after treatment with WFC; VitB, patients before treatment with vitacoenzyme; VitA, patients after treatment with vitacoenzyme; **, P < 0.01, compared with Vit

As shown in Table 4, effective rate of alleviating clinical symptom in the WFC group (86.67%) was higher than in the Vit group (23.33%) (total effective rate for alleviating clinical symptom = (total clinical symptom score before treatment-total clinical symptom score after treatment)/before treatment × 100%), indicating that WFC could dramatically improve clinical symptoms.

**Table 4:** Comparison of clinical symptom cumulative score

Group	*n*	Before treatment	After treatment	Effective rate (%)	t	*P*
WFC	30	15.32 ± 5.71	6.27 ± 2.19	86.67	23.231	< 0.01
Vit	30	15.92 ± 5.43	13.69 ± 4.12	23.33		

WFC, Weifuchan Group; Vit, Vitacoenzyme Group;

### The taxonomic composition of intestinal microbiome

Sixty-six feces samples from all participants (28 samples from patients before and after treatment with WFC, 28 samples from patients before and after treatment with Vit drug, and 10 samples from healthy volunteers) were collected and sequenced the variable region V3 of the 16SrRNA using the Illumina MiSeq platform. A total 6,122,474 sequences ranging from 86,389 to 102,665 sequences per sample (mean = 92,764.758; median = 92,591) were obtained after quality control analyses. From these data, we identified a total of 62,453 OTUs.

**Fig. 3:**
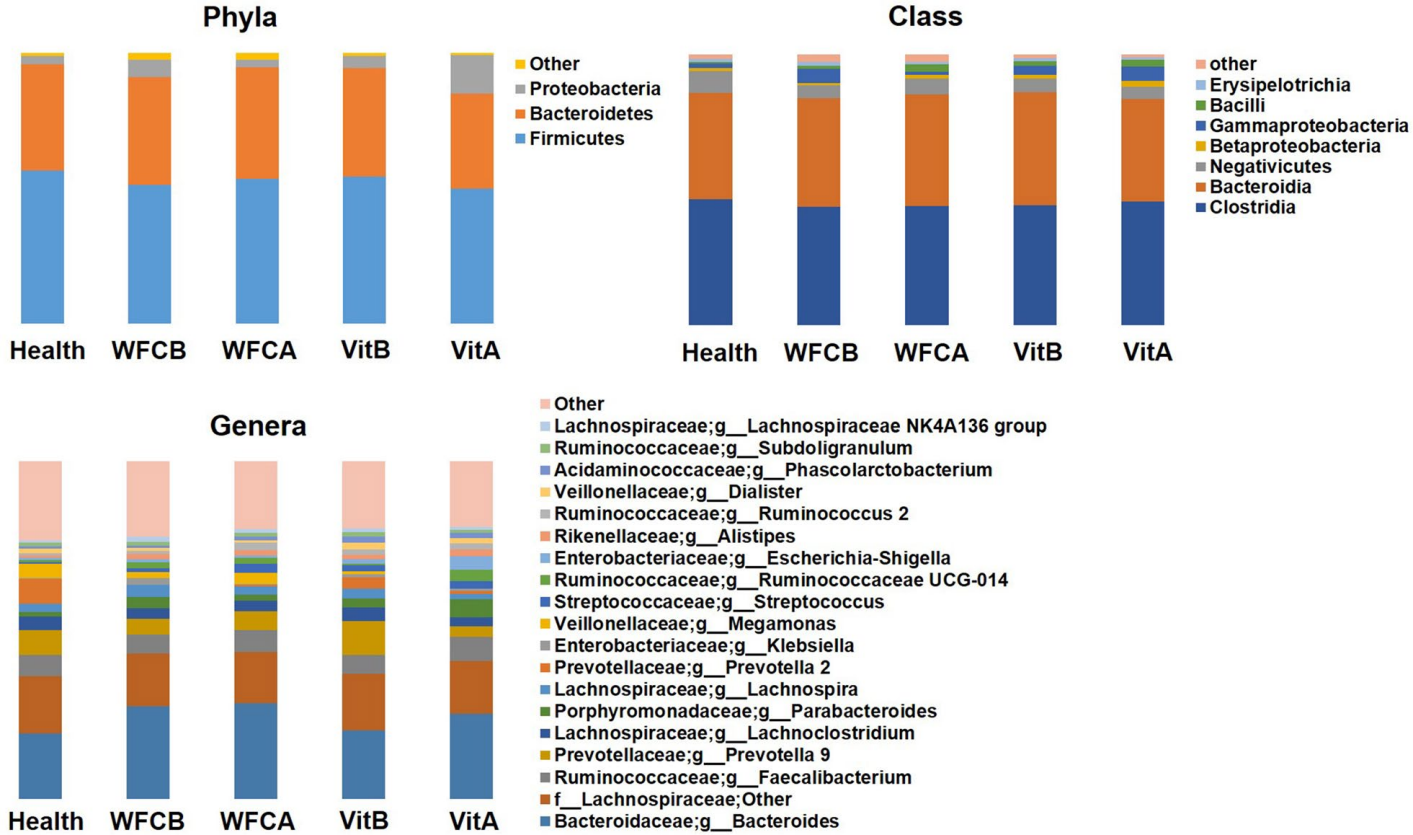
Microbial profiles (mean relative proportion) of most abundant (> 1%) phyla, class and genera by comparison for groups. Health: healthy volunteers; WFCB: PLGC patients before treatment with WFC; WFCA: PLGC patients after treatment with WFC; VitB: PLGC patients before treatment with vitacoenzyme; VitA: PLGC patients after treatment with vitacoenzyme

The intestinal microbiomes across all 66 samples included sequences that corresponded to 3 dominant (> 1.00%) Phyla: *Firmicutes*(52.74%), *Bacteroidetes* (39.10%) and *Proteobacteria* (6.44%). These Phyla comprised 7 dominant (> 1.00%) class and 19 dominant (> 1.00%) genera. Top 6 dominant (> 3.00%) genera were *Bacteroides* (24.51%), *Lachnospiraceae* (unclassified) (15.87%), *Faecalibacterium* (6.25%), *Prevotella 9*(6.07%), *Lachnoclostridium* (3.37%), and *Parabacteroides*(3.00%). The mean relative proportion of dominant (> 1%) phyla, class and genera in the five groups were shown in Fig. 3. All these genera are commonly found in the feces of individuals with and without PLGC, although in different proportions [22–26].

A variable number of OTUs from these 19 dominant (> 1.00%) genera were included in the core microbiome, which could comprise the stable and consistent members and associations in the whole community [27, 28]. The least stringent definition of the core (presence in at least 50% of the samples) identified 127 OTUs of commensal and pathogenic bacteria; while a more stringent definition (presence in at least 95% of the samples) included 8 OTUs of the following genera: *Bacteroides, Lachnospiraceae* *(unclassified)*, *Faecalibacterium*, *Lachnoclostridium*, *Parabacteroides, Streptococcus, Escherichia-Shigella,* and *Lachnospiraceae.* Pathogenic representatives from *Bacteroides, Lachnospiracea, Faecalibacterium*, *Lachnoclostridium* and *Streptococcus* genera have been consistently associated to gastric cancer [29–32]. Unlike the above 5 dominant genera, *Parabacteroides* could be more related to the gastric and intestinal disease such as dyspepsia [33], and *Escherichia-Shigella and Lachnospiraceae NK4A136 group* were involved in gastrointestinal inflammation and immunity [34, 35].

### Univariate, receiver operating characteristics (ROC) curve

**Fig. 4:**
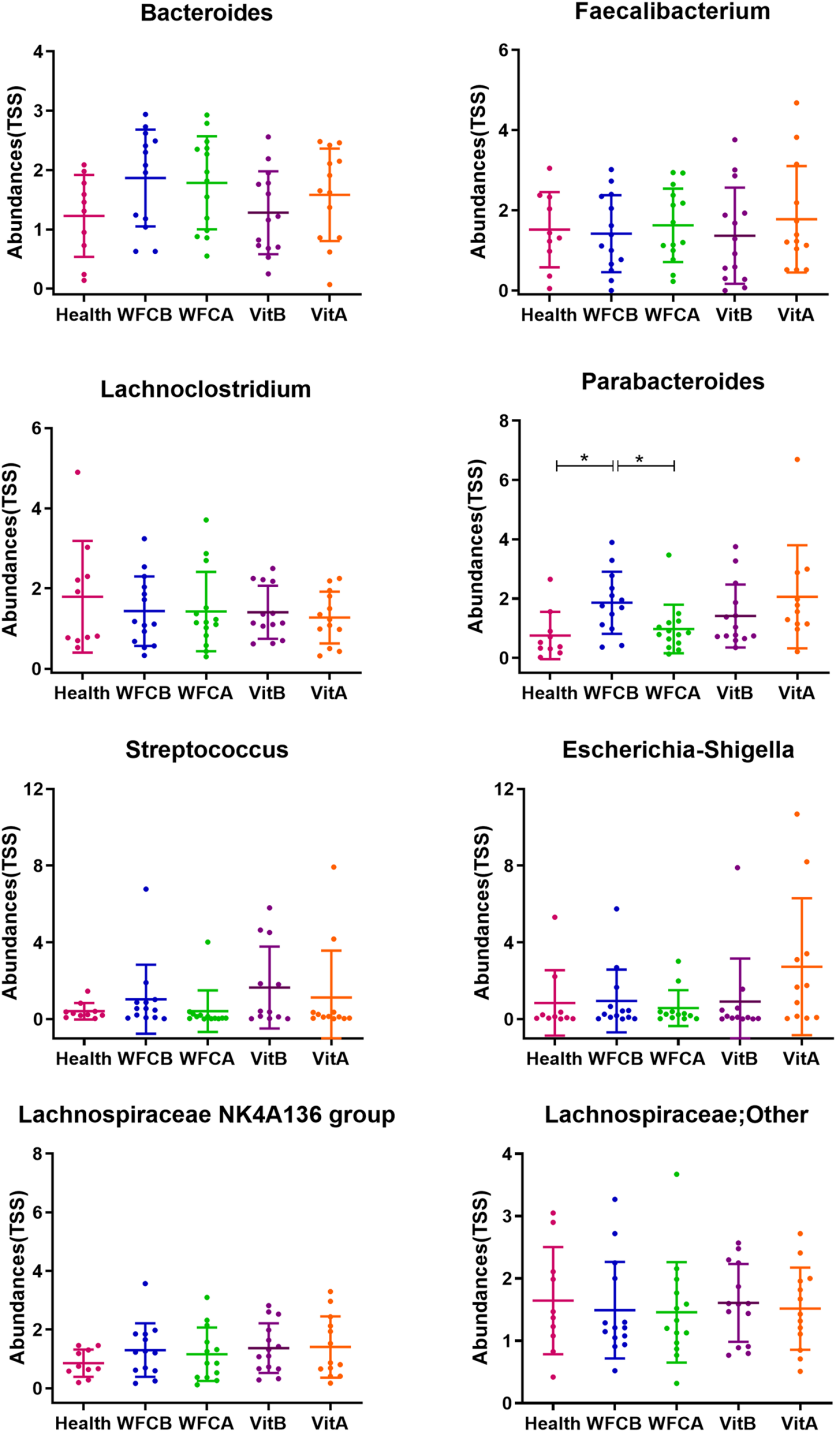
Comparisons for bacterial abundance. Bacterial relative abundance differences were observed for 8 core genera in each group. TSS: Total -sum normalization. *, p < 0.05

**Fig. 5:**
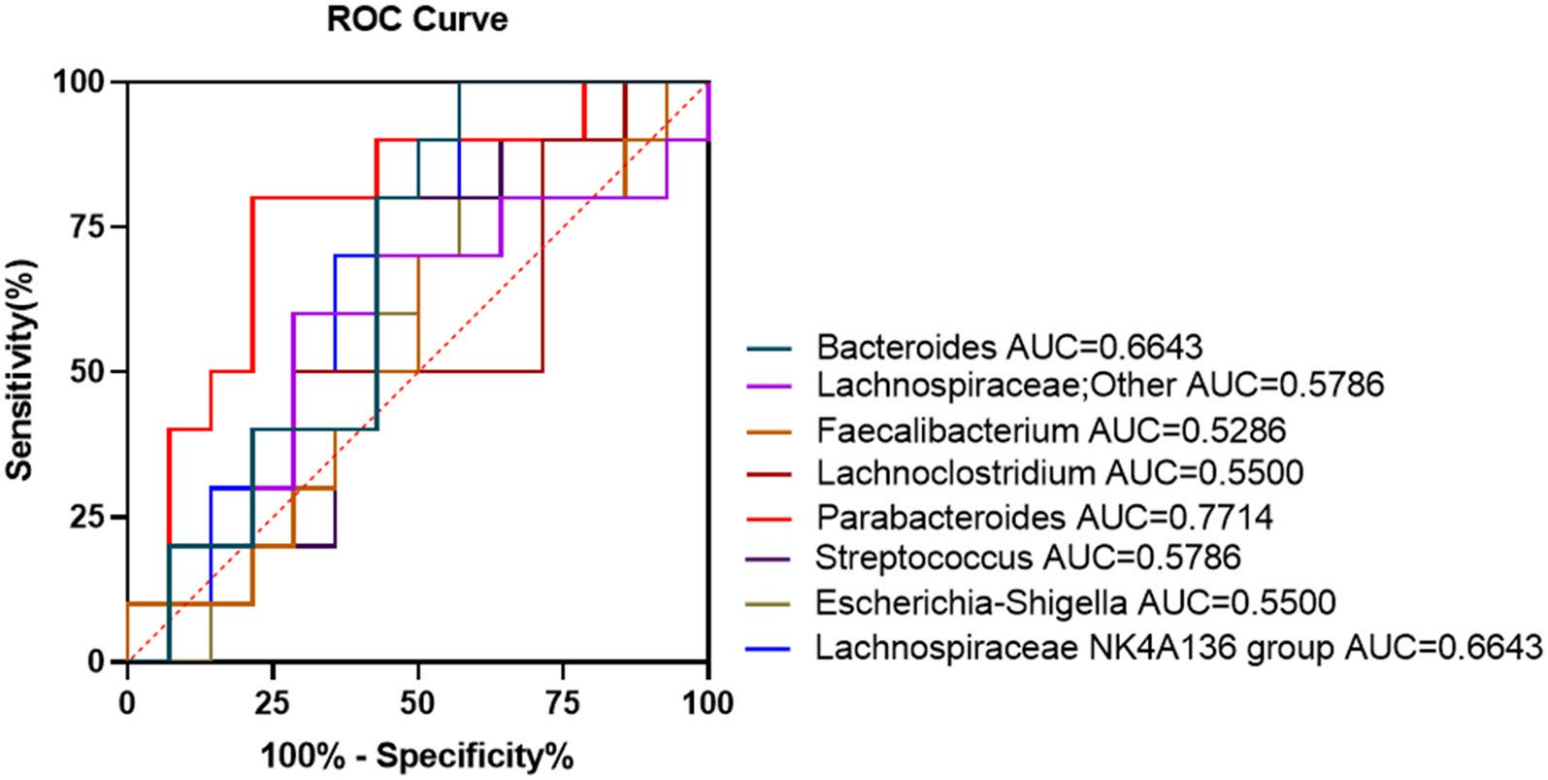
Receiver operating curve analysis for selected microbial biomarker of PLGC. 8 selected microbial biomarkers of PLGC were tested by ROC analysis

As shown in Fig. 4, microbial abundances of all the eight dominant bacterial genera was compared between various groups based on the ANOVA, and between WFCB vs WFCA and VitB vs VitA by the paried t-test. All candidates, except for *Parabacteroides*, were not significantly different between groups. Compared with Health group, the relative abundance of *Parabacteroides* was significantly higher in WFCB group. On the other side, abundance of *Parabacteroides* declined observably in WFCA group. This decrease was not observed in the Vit group. The arithmetic mean ± standard deviation of the relative abundance (raw count number reads) were 0.76 ± 0.76 (14729.04 ± 14767.28) for Health group, 1.86 ± 1.01(35775.48 ± 20532.81) for WFCB group and 0.98 ± 0.79 (19062.95 ± 15363.4) for WFCA group. 8 candidate genera were selected to analyze ability to assess PLGC using ROC curve. The results were shown in the Fig. 5 and in Table 5. The area under the curve (AUC) was > 0.7 and p value < 0.05 only for *Parabacteroides (AUC=0.7714,p value=0.026)*. The other 7 candidate genera were all < 0.7 in AUC, suggesting that *Parabacteroides* may be an important candidate in the assessment of PLGC development and the therapeutic effect of WFC on PLGC.

**Table 5:** The best cut off points for selected microbial candidates of PLGC

Out	Area	Lower bound	Upper bound	Cut off value	Sensitivity	Specificity	*p* value
*Bacteroides*	0.66	0.44	0.88	3.494	0.43	1	0.178
*Lachnospiraceae; Other*	0.58	0.33	0.82	89550.33	0.93	0.2	0.520
*Faecalibacterium*	0.53	0.29	0.77	96980.75	0.21	0.9	0.815
*Lachnoclostridium*	0.55	0.31	0.79	19030.36	0.71	0.5	0.682
*Parabacteroides*	0.77	0.57	0.97	18354.22	0.79	0.8	**0.026**
*Streptococcus*	0.58	0.34	0.81	4847.02	0.57	0.8	0.520
*Escherichia-Shigella*	0.55	0.31	0.79	3969.2	0.43	0.8	0.682
*Lachnospiraceae NK4A136 group*	0.66	0.45	0.88	11207.62	0.43	1	0.178

Only *Parabacteroides* have statistical difference in ROC curve

## Discussion

Gastric cancer develops through a multistep process triggered by *H. pylori* and progression from superficial gastritis to atrophic gastritis, IM, and dysplasia [36]. Atrophy, IM or dysplasia is considered as PLGC and require accurate surveillance programs. PLGC belong to the “stomach distension” and “epigastralgia” category in traditional Chinese medicine. WFC is a clinical effective prescription for body discomfort including distension and fullness of the stomach, belching and poor appetite, constipation or diarrhea, lassitude and weakness, dizziness and emaciation, sallow complexion. In this study, the results showed that WFC could significantly improve clinical symptoms and gastric mucosa pathology, especially atrophy and IM.

The etiology of *H. pylori*-positive has been well described over the past few decades [37], but *H. pylori* eradication cannot completely eliminate the recurrence risk of gastric cancer [38], suggesting there is another factor affecting the progression of gastric cancer. It has been reported that the alternations of fecal microbiota involved in the process of *H. pylori*-related gastric lesion progression [39]. Resident microbes can induce inflammation, leading to cell proliferation and altered stem cell dynamics, which can lead to alternations in DNA integrity and immune regulation and promote carcinogenesis [40]. In this study, we observed gut microbes alternation in PLGC population with *H. pylori* negative and the effect of WFC on this population. The result found that 8 types of microbes may dominate the bacterial community of PLGC at genus level. Further analysis found that only the abundance of *Parabacteroides* was significantly different in Health group vs WFCB group and in WFCB group vs WFCA group. AUC was > 0.7 for *Parabacteroides* in ROC analysis which could effectively distinguish PLGC patients from healthy individuals, with sensitivity of 0.79 and specificity of 0.8, suggesting the importance of *Parabacteroides* in PLGC occurrence.

*Parabacteroides* genera belong to the *Bacteroidetes* phyla and the *Porphyromonadaceae* family. Among the gut *Parabacteroides*, *Parabacteroides Distasonis* (*P. distasonis*) is defined as one of the 18 core members in the gut microbiota of humans and thought to have important physiological functions in hosts [41]. Results from animal studies proved the protective role of *P. distasonis* in colonic tumorigenesis and maintenance of intestinal epithelial barrier in AOM-treated mice [42]. A study from 736 American Gut Project sample found that the abundance of *P. distasonis* is relatively lower in patients with obesity, inflammatory bowel diseases, nonalcoholic fatty liver, and multiple sclerosis [43–45]. However, some studies found the relative abundance of *Parabacteroides* are increased in heart diseases [46], leukemia [47, 48] and early hepatocellular carcinoma [49]. The phenotype of cancer cachexia is associated with increased levels of *Parabacteroides* [50]. The studies suggested *Parabacteroides* may perform many biological functions in the human body.

Recent evidence from in *vivo* and *vitro* confirmed that *P. distasonis* possessed a strong ability to transform primary bile acids into secondary bile acids and enhancing the level of succinate in the gut [26, 51]. The gut microbiota and the bile acid pool played pivotal roles in maintaining intestinal homeostasis. Interplay between bile acid and the gut microbiota promoted gastrointestinal carcinogenesis [52]. It was also reported that bacterial metabolites, including secondary bile acid, had the potential to cause direct DNA damage or to provoke inflammation, which in turn promoted carcinogenesis [53]. Bile acids could promote gastric IM by upregulating CDX2 and MUC2 expression via the FXR/NF-kB signaling pathway [54]. Succinate, the intermediates of the mitochondrial pathway known as the Krebs cycle, had extensive evidence for “non-metabolic” signaling functions or metabolic reprogramming leading to altered immune cell and transformed cell function in the initiation of carcinogenesis [55, 56]. Additionally, *Parabacteroides* in the gut could use type VI secretion systems (T6SSs) to antagonize symbiotic gut *E. coli*, facilitating colonization and cancer progression [57]. To sum up, *Parabacteroides* are multifunctional bacteria in the human gut and have the potential capacity to promote gastric carcinogenesis. In our study, the relative abundance of *Parabacteroides* in PLGC group was significantly high before treatment with WFC. In contrast, a decreased abundance of *Parabacteroides* was observed in PLGC group after treatment with WFC. The effect of WFC on gut *Parabacteroides* corresponded with the results of gastric histology in PLGC. Accumulating data suggested that gut microbiota had a role in the etiology of several types of cancer, including gastric cancer. However, data about intestinal microbiota correlation with PLGC were not enough. Further studies on the alteration of intestinal flora in PLGC development are needed. It has been reported that *Parabacteroides* play a predominant role in anti-obesity effects [58], but short of evidence for its effects on gastric cancer. Our research explore the relationship between PLGC pathology variation and *Parabacteroides* in clinical trial, which maybe reveal a novel and potential mechanism and will provide help for further studies regarding the mechanisms of action of WFC on chronic gastric disease. In our previous research, we found that WFC could improve histopathological changes of gastric mucosa of PLGC in rats induced by N-methyl-N'-nitro-N-nitrosoguanidine partly by inhibiting MAPK signaling pathway to increase pepsin secretion [12]. In this study, the results suggested that WFC could inhibit inflammation of gastric mucosa by regulating the abundance of gut microbiota. But further studies are needed to investigate causal relationships between WFC and intestinal flora in PLGC. The study had few limitations including the limited sample size, especially for collected feces for intestinal flora. Larger sample sizes are needed in order to confirm the role of *Parabacteroides* in the development of PLGC.

## Conclusions

This study suggested WFC slowed down PLGC, which could be related to *Parabacteroides* abundance variation. The results will help elucidate the effects of WFC on PLGC and provide a treatment method for PLGC.

## Data Availability

The datasets used and/or analyzed during the current study are available from the corresponding authors on reasonable request.

## Abbreviations

PLGC: Precancerous lesions of gastric cancer
WFC: Weifuchun
CAG: Chronic atrophic gastritis
GPL: Gastric precancerous lesions
IM: Intestinal metaplasia
CI: Chronic inflammation
AI: Acute inflammation
CFDA: China food and drug administration
Health: Healthy volunteers
WFCB: Patients before treatment with WFC
WFCA: Patients after treatment with WFC
VitB: Patients before treatment with vitacoenzyme
VitA: Patients after treatment with vitacoenzyme
OTUs: Operational taxonomic units
